# A case report of monkeypox as a result of conflict in the context of a measles campaign

**DOI:** 10.1016/j.puhip.2022.100312

**Published:** 2022-09-12

**Authors:** E.L. Jarman, M. Alain, N. Conroy, L.A. Omam

**Affiliations:** a Reach Out Cameroon, Buea, Southwest Region, Cameroon; b School of Public Health, University College Cork, Ireland; c Department of Public Health and Primary Care, University of Cambridge, UK

**Keywords:** Monkeypox, Measles, Southwest Cameroon

## Abstract

**Introduction:**

Northwest and Southwest Cameroon suffer with ongoing conflict, associated with internal displacement of communities into bushland, violence and destruction of the health system.

**Case presentation:**

During a measles immunisation and surveillance campaign, following a measles outbreak, a 14-year-old boy was identified as having fever and a rash. This developed following close contact with a giant forest rat. He was diagnosed with monkeypox and this was confirmed by PCR. He was treated with oral cloxacillin and topical tetracycline for superadded bacterial skin and eye infections, and isolation policies were put in place. His rash improved over 7 days, when it scabbed over and his fever settled.

**Discussion:**

Recent displacement into a bush settlement away from agricultural opportunities increased his family’s reliance on bush meat. Displacement away from established surveillance systems increased the risk of undetected transmission. This is the second confirmed case of Monkeypox in Cameroon in the last year, and the first in the Southwest region.

**Conclusion:**

Conflict led to the breakdown of surveillance systems, a lack of health personnel, destruction of health facilities and displacement of communities, which raised the risk of monkeypox outbreaks within Northwest and Southwest Cameroon. Surveillance for monkeypox is challenging due to clinical similarity to chickenpox. There is a risk of emergence in new regions with suitable hosts. The factors underlying the establishment of monkeypox infections in new regions are multifactorial and require a strong public health response for prevention and control. A OneHealth approach to emerging infectious diseases is essential.

## 1 Introduction

A conflict within the regions of Northwest and Southwest of Cameroon has led to significant internal displacement within some communities. Affected populations have fled into neighbouring bushland, as well as into other regions of Cameroon and to neighbouring Nigeria. The Reach Out Cameroon team consisting of 1 doctor, 2 nurses and 40 community health workers were running a community based surveillance and primary health care project in the area. In August 2019, a measles outbreak was declared in the Southwest region [1]. As part of the surveillance response, a child with a fever and rash was flagged to the Reach Out Cameroon team. The team had 42 cases of outbreak-associated measles reported to them over a 9 month period, in a population of approximately 55,000 people [1]. Monkeypox is a zoonosis with similar symptoms to smallpox and chickenpox. It is endemic in parts of West and Central Africa.

## 2 Case presentation

An otherwise healthy 14-year-old male presented to the District Hospital in Ekondo Titi, Southwest region, with fever and a pruritic vesiculopustular rash, which originated on his left knee and scalp. It then spread to his face, chest, arms and legs, including his soles and palms. The rash developed over 1–2 days. A prodrome of fever and headache lasted for 1 day, with an intermittent temperature of 38.5° associated with rigors, after which the rash developed. There were painful lesions in his mouth, as well as groin pain, exacerbated by movement. He also reported sore, bilaterally red eyes with no visual changes and no discharge. His mother reported that he had chickenpox as a small child.He regularly hunted for the family, catching, preparing and eating giant forest rats. He had caught a forest rat one week previously and prepared it prior to himself and his family eating it.

**Fig. 1:**
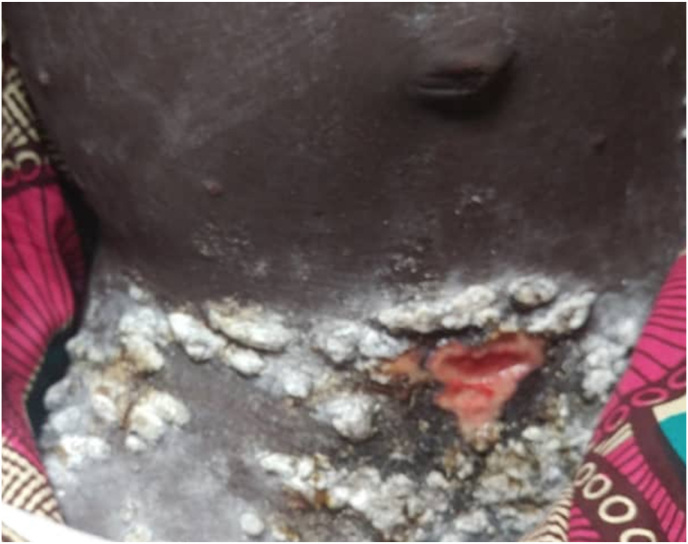
Suprapubic and Para inguinal lesions at diagnosis.

On examination, he had multiple pustules located all over his body, including his scalp, soles of his feet and palms of his hands. They were not evenly distributed but did cover his entire body, and were more prominent on his head, hands and feet. The lesions were pedunculated and similar to each other, and were not pleomorphic. He had palpable, non-tender small submental and submandibular lymph nodes, and larger, tender inguinal lymph nodes bilaterally. Below his umbilicus, multiple pustular lesions converged to form an ulcer (Fig. 1). He had bilateral conjunctival injection. The rest of his examination was normal.

Two lesion swabs were taken from the suprapubic lesion, and a lesion on his hand which were both positive for orthopox and monkeypox on PCR. Whole blood sample was negative for orthopox and monkeypox virus on PCR. His malaria test and HIV test were negative.

He was initially treated for chicken pox with calamine lotion and paracetamol. Upon diagnosis of monkeypox, he was treated with oral cloxacillin 500 mg four times daily for 7 days to treat a likely superadded bacterial skin infection in view of the rigors and tetracycline 1% eye ointment for 5 days to cover for bacterial conjunctivitis. This was tolerated well. His lesions were cleaned by nursing staff. He was isolated and his mother and health care workers caring for him were taught the importance of hand hygiene and infection control policies. There was no available masks or aprons, so health care workers were encouraged to use gloves and to wash their hands thoroughly. His lesions scabbed over after 7 days. He completed his course of antibiotics and was discharged home when his final lesion had scabbed over, and his ulcer had healed. There were no secondary cases identified despite contact tracing of 24 individuals, including family members and healthcare professionals. These were followed up by phone for 2 weeks.

## 3 Discussion

This case of monkeypox is noteworthy, as it represents introduction of the disease into a region where it had not been described previously, in a patient who was exposed to risk because of internal displacement. The area in which this case occurred is typically an agricultural area, with large amounts of subsistence farming.However, due to the conflict families have been forced to abandon their farms, and this has led to an increased reliance on hunting, and bushmeat. In short, this case describes some of the conditions that can facilitate the establishment of a zoonotic disease in new population.

In 2018, there was 1 confirmed case of monkeypox and 15 suspect cases in the neighbouring Northwest region region. The confirmed case was a 20-year-old male. He presented with a vesiculo-pustular rash and lymphadenopathy. Monkeypox remains a rare disease in this district as well [2].

Monkeypox is caused by an orthopox virus, similar to smallpox. It is a zoonosis which has been documented within the Congo basin. The largest numbers of cases have occurred in the Democratic Republic of Congo, with smaller numbers of cases across West and Central Africa [3]. There are two genomic variants, a West African variant and a Central African clade, with varying mortality rates. The Central African variant has a fatality rate of 11–15% [3]. It has been mostly reported in rural areas where there is contact with potential reservoirs, such as African rodents and monkeys [2]. Transmission is by direct contact with these animals, consumption of bush meat or close contact with an infected person. This disease is considered an emerging disease. Smallpox vaccination is at least 85% effective at preventing monkeypox [4], and the cessation of the use of smallpox vaccine makes outbreaks of monkeypox more likely.

Although this case was identified as part of a measles campaign, the greatest diagnostic challenge is often distinguishing monkeypox from chicken pox. In this case, the negative whole blood PCR presented an extra diagnostic challenge.

## 4 Conclusion

This case demonstrates a classic presentation of monkeypox within a complex public health context. This is the first case of monkeypox recorded in the Southwest region of Cameroon, and there has been one further case identified in the Northwest since the start of this conflict in 2016 [2]. Conflict has historically led to the breakdown of surveillance systems, and displacement of communities [5]. This case highlights the importance of surveillance systems for epidemic prone diseases within conflict zones and how the shift in the balance of risk, as communities are forced to abandon their normal livelihoods and activities and adapt to new surroundings in conflict situations, can lead to emerging diseases for these geographical locations. Key public health lessons from cases like this are 1) The difficulty of surveillance for monkeypox, due to its clinical similarity to chickenpox. 2) Monkeypox can emerge in regions with suitable hosts, and extrinsic factors can affect its stability in any given region. 3) The factors underlying the establishment of monkeypox infections in new regions are likely to be multifactorial, and require a strong public health response to prevent and control them. A OneHealth approach to emerging infectious diseases is essential.

## Ethical approval

Not applicable.

## Consent for publications

Written informed consent was obtained from the patient’s legal guardian(s) for publication of this case report and any accompanying images. A copy of the written consent is available for review by the Editor-in-Chief of this journal.

## Funding

Reach Out N.G.O staff identified, managed and wrote this case report. The organisation is funded by 10.13039/100006641UNICEF to conduct health activities in this area.

## Declaration of competing interest

The authors of the above paper declare no conflict of interests.
